# Spatio-Temporal Differences in Dystrophin Dynamics at mRNA and Protein Levels Revealed by a Novel FlipTrap Line

**DOI:** 10.1371/journal.pone.0128944

**Published:** 2015-06-17

**Authors:** Frederique Ruf-Zamojski, Vikas Trivedi, Scott E. Fraser, Le A. Trinh

**Affiliations:** 1 California Institute of Technology, Biological Imaging Center, Beckman Institute, Division of Biology, Pasadena, California 91125, United States of America; 2 California Institute of Technology, Department of Bioengineering, Pasadena, California 91125, United States of America; University of Minnesota Medical School, UNITED STATES

## Abstract

Dystrophin (Dmd) is a structural protein that links the extracellular matrix to actin filaments in muscle fibers and is required for the maintenance of muscles integrity. Mutations in Dmd lead to muscular dystrophies in humans and other vertebrates. Here, we report the characterization of a zebrafish gene trap line that fluorescently labels the endogenous Dmd protein (Dmd-citrine, *Gt(dmd-citrine)^ ct90a^*). We show that the Dmd-citrine line recapitulates endogenous dmd transcript expression and Dmd protein localization. Using this Dmd-citrine line, we follow Dmd localization to the myosepta in real-time using time-lapse microscopy, and find that the accumulation of Dmd protein at the transverse myosepta coincides with the onset of myotome formation, a critical stage in muscle maturation. We observed that Dmd protein localizes specifically to the myosepta prior to *dmd* mRNA localization. Additionally, we demonstrate that the Dmd-citrine line can be used to assess muscular dystrophy following both genetic and physical disruptions of the muscle.

## Introduction

Dystrophin (Dmd) is an essential structural protein in the Dystrophin Associated Protein Complex (DAPC) of skeletal muscles that links the extracellular matrix to the cytoskeletal actin filaments of the myofibril [1, 2]. Mutations in components of the DAPC result in muscular dystrophy, a condition characterized by progressive muscle weakness and degeneration. Dmd mutations are the cause of approximately 90% of all human forms of muscular dystrophies. In particular, DMD is the gene that is mutated in most cases of Becker Muscular Dystrophy and Duchenne Muscular Dystrophy (BMD and DMD, respectively) [3–6]. DMD is an X-linked recessive, fatal disorder. It has been estimated that 1 in 3,500 to 5,000 boys suffers from DMD [7, 8], and that approximately three to six of every 100,000 live births are affected by BMD [9, 10].

Several vertebrate models have been developed to study DMD [11], ranging from mdx mice [12, 13] and dystrophic golden retriever dogs [14], to DMD-deficient cats [15], and mutant zebrafish [16–18]. Several zebrafish alleles of *dmd* mutations were identified by the Tubingen screen [16], including *dmd* ^*tm90c*^, *dmd* ^*ta222a*^ and *dmd* ^*tj7*^. Twelve additional alleles have been identified and referenced by the Zebrafish Mutation Project conducted at the Sanger Institute [19]. The dystrophin-deficient zebrafish *dmd* ^*ta222a*^ (*Sapje* mutant) has a nonsense mutation in exon 4 of *dmd* [17]. The *Sapje-like* mutant was isolated more recently, and carries a *dmd* splice site mutation within exon 62 [18]. The Sapje and Sapje-like alleles recapitulate the muscle degeneration phenotypes observed in humans [17, 18], providing a good model for the study of muscular dystrophies [20]. These genetic models have provided many insights into the molecular lesions that can lead to muscular dystrophy; however, real-time monitoring of Dmd expression and localization *in vivo*, in order to assess both wildtype muscle development and progression of muscle dystrophy at the cellular and protein level, remains a challenge. The challenge results, in part, from the large size of Dmd, a 427 KDa protein that is encoded by 79 exons [21, 22]. The Dmd protein is composed of four major domains: 1) an actin-binding N-terminal domain, 2) a large array of 24 spectrin-repeats that form triple-helical coiled coils, 3) a cysteine-rich domain composed of EF-hands and a ZZ zinc finger motif and 4) a C-terminal domain that binds components of the DAPC [23, 24]. In addition, four proline-rich hinge domains link the spectrin-repeats in the Dmd protein. These hinge domains have been shown to influence muscle maturation and maintenance, as well as the structures of the myotendinous junction and of the neuromuscular synapses [25]. This modular organization of the Dmd protein into large, well-conserved protein domains suggests that a compact insertion, such as a fluorescent protein, might be added by genetic engineering without disrupting the function of Dmd.

Immunocytochemical localization of Dmd protein in skeletal muscles shows a reproducible progression with development. In humans, Dmd protein is initially detected in the cytoplasm of skeletal muscles, but it is later localized to the plasma membrane after 10 weeks of gestation [26, 27], and is localized mainly at the neuromuscular junction upon birth. It has long been suggested that different isoforms account for the differential localizations of Dmd, as different staining patterns have been observed using different antibodies against specific domains of Dmd [27]. The differential localization of Dmd can be recapitulated in primary muscle cell cultures, as Dmd is detected in the sarcoplasm during early myoblast fusion, and at the cell membrane in more differentiated myotubes [28]. In zebrafish embryos, Dmd protein has been reported to be localized to the myosepta [18, 29–35]. In adult zebrafish there is a shift in Dmd localization (starting around 7 days post fertilization) to a more widespread sarcolemmal expression [36, 37] and to the muscle cell membranes [37].

Vertebrate skeletal muscle arises from the paraxial mesoderm, which becomes segmented into the repeated structure of the somites, forming sequentially in an anterior to posterior pattern (reviewed in [38]). In zebrafish, the first pair of somites is formed at 10.5 hours post-fertilization (hpf). The next 5 pairs appear every 20 minutes thereafter, followed by the remaining 24 pairs every 30 minutes at 28.5°C [39, 40]. Each somite is separated from its rostral and caudal neighbors by a sheet of extracellular matrix, the vertical myoseptum, and each somite is divided into dorsal and ventral muscle masses by another sheet of matrix that forms the horizontal myoseptum. The muscle fibers that arise from each somite are thought to use the myosepta for attachment. Dmd appears to play a role in this attachment [17], as failure of these attachments to form during the development of *sapje* mutant embryos, results in muscular dystrophy phenotypes [41]. Although multiple studies demonstrate that Dmd is an important player in muscle development, structure, maintenance and signaling, all analyses of Dmd localization have been based on fixed specimens. For example, in the live imaging study of muscle fiber attachment in *Sapje* mutants [35], the Dmd protein dynamics could not be assessed. As muscular dystrophy is a progressive disorder, the ability to follow Dmd and the progression of the disease in real time would be informative; however, there has been no animal model with a fluorescently tagged Dmd protein that would permit such studies.

Here, we characterize a gene trap line in which full-length Dmd is fused to the fluorescent protein Citrine (*Gt(dmd-citrine)* ^*ct90a*^). We demonstrate that the fusion protein (Dmd-citrine) and transcript (*dmd-citrine*) recapitulate the endogenous expression of the Dmd protein and transcript. Interestingly, we observe a spatial difference of *dmd* transcript localization during development that is not reflected at the protein level. Real-time analysis in *Gt(dmd-citrine)* ^*ct90a*^ embryos show that, unlike its transcript, Dmd protein is expressed at the myosepta as soon as it is detected, and this protein localization becomes more defined and sharper as the skeletal muscle develops. Finally, we illustrate that the *Gt(dmd-citrine)* ^*ct90a*^ line can be used to assess muscle phenotypes *in vivo*.

## Materials and Methods

### Zebrafish

This study was carried out in strict accordance with the recommendations in the Guide for the Care and Use of Laboratory Animals of the California Institute of Technology. The protocol was approved by the Institutional Animal Care and Use Committee (IACUC) of the California Institute of Technology (Permit Number: 1227). Wild-type embryos were obtained from AB and TL strains. *Gt(dmd-citrine)* ^*ct90a*^ transgenic embryos were obtained from a screen performed in the laboratory [42]. Adult fish were maintained as described in [43].

### RNA injections and morpholino experiments

For RNA microinjection, 2.3nL of a 50ng/μL mRNA encoding for membrane-mCherry and H2B-cerulean were injected into 1-cell stage embryos. The plasmids used were pCS-H2B-cerulean and pCS-membrane-mCherry [44]. They were linearized with NotI, purified using a Qiagen nucleotide removal spin column and used as template for *in vitro* transcription with the Ambion Message Machine kit (Life Technology, catalogue #AM1340). *Gt(dmd-citrine)* ^*ct90a*^ zebrafish embryos were injected at the 1-cell stage with 10ng of *dmd* morpholino antisense oligonucleotide (*dmd* AS2 morpholino from [45], sequence: 5'-TTGAGTCCTTTAATCCTACAA TTTT-3’) or 10ng of inverted dmd morpholino / control morpholino (AS2 inverted from [45], sequence: 5'- TTTTAACATCCTAATTTCCTGAGTT-3’). Morpholinos were diluted following manufacturer instructions (Gene Tools). Embryos were then raised, at 28.5°C, to the desired stage for analysis.

### Immunohistochemistry

Embryos were raised to the stage required, dechorionated, and anesthetised in 0.01% tricaine before being fixed in 4% Paraformaldehyde (PFA). Following fixation, embryos were pre-incubated in Phosphate Buffered Saline (PBS) with DMSO and Triton (PBDT; 1X PBS, 0.1% Triton-100, 1% Bovine Serum Albumin (BSA), 1% DMSO) for 1h at room temperature. Incubation with the primary antibody was performed overnight at 1:200 dilution in PBDT, followed by washing and incubation with a secondary Alexa- coupled antibody (546nm, Invitrogen) for 4h at room temperature (RT) in PBDT. Primary antibodies used were: anti-Dmd (Mandra-1, Developmental Studies Hybridoma Bank (DSHB)), anti-Myosin heavy chain fast isoforms (F59, DSHB), anti-Myosin heavy chain slow muscle fibres (S58, DSHB), anti-Tropomyosin (CH1, DSHB), anti-Laminin (Sigma, catalogue #L9393), and anti-GFP to recognize Citrine (Torrey Pines Biolabs, catalogue #TP-401).

### Imaging, reconstructions and analysis

Imaging was performed on a Zeiss LSM510 inverted confocal microscope. Embryos were placed into agarose moulds [44] and imaged with the following lasers: 514nm to excite Citrine; 543nm to excite Bodipy-TR, Alexa-546 and membrane-mCherry; 458nm to excite H2B-cerulean. Images were taken using a water-immersion 40X 1.1NA Apochromat objective using the Zeiss LSM software and time-lapse imaging was performed using the Zeiss multi-time macro. To permit accurate comparisons, all embryos in a given experiment were imaged using the same laser power, gain detector settings and scanning speed. For live imaging, BODIPY-TR (Life Technology, catalogue #C34556) was used as a counterstain. 3D reconstructions and projections were performed using Imaris 6.4 (Bitplane). Custom written codes in MATLAB 2013b were used to quantify the number of pixels and their intensities. The kymograph was generated by assigning all the pixels of a given time point one single color.

### Chromogenic *in situ* hybridization (ISH)

Chromogenic ISHs were performed and imaged as described in [46]. A digoxigenin (DIG) probe was synthesized using a plasmid template that contained a 2Kb fragment of the 3’ end of the *dmd* gene. Embryos were dechorionated and fixed in 4% PFA overnight at 4°C before being dehydrated in a series of methanol and phosphate buffered saline with Tween (PBST; 1X PBS, 0.1% Tween-20). Proteinase K was used to permeabilize embryos before washing and pre-hybridized at 70°C for 1h. Hybridization was performed overnight at 70°C and DIG probe was detected with 1:5,000 anti-DIG alkaline phosphatase. Stained embryos were imaged with both wide-field and confocal microscopes. To improve the 3D localization and resolution of the ISHs, we used the technique described by Trinh and colleagues [46] to detect the far-red fluorescence of the NBT/BCIP chromogenic stain using a 510 LSM Zeiss confocal microscope.

### Western-blotting

Batches of 20 embryos (72 hpf) were dechorionated and pooled together per condition in egg water (0.6g/L aquarium salt in water, 0.01mg/L methylene blue) and 0.01% Tricaine. Anesthetized embryos were de-yolked in calcium-free Ringer’s solution (116mM NaCl, 2.9mM KCl, 5mM HEPES pH 7.2), rinsed two times in fresh calcium-free Ringer’s solution before being put into Tris buffer saline with NP40 (1% NP40, 150mM NaCl, 50mM Tris-HCl pH8) with anti-proteases (Roche, Complete Mini, catalogue #04693159001). After 30 minutes on ice and vortexing, the samples were centrifuged at top speed for 15 minutes at 4°C. The supernatant was collected and used for Western blotting. Standard Western-blotting techniques were followed using gradient 4–15% Tris-Acetate gels (Bio-Rad). Membranes were incubated in primary antibody, followed by washing and incubating in anti-mouse-conjugated goat horseradish peroxidase (HRP) secondary antibody (1: 10,000). Dmd was detected using the Mandra-1 antibody (DSHB, 1:1,000) and Tubulin using anti-alpha Tubulin, clone DM1A (Sigma, 1:3,000). We detected a single band at greater than 180kDa in the Westerns, indicating that this antibody may not detect all isoforms of Dmd. The blots were finally incubated in Western Lightning Chemiluminescence Reagent (Perkin Elmer), as suggested by the manufacturer, and exposed to film.

### Heat-shock treatment

Batches of 20 6-somite stage *Gt(dmd-citrine)* ^*ct90a*^ embryos were dechorionated and pooled together in 0.5mL egg water (0.6g/L aquarium salt in water, 0.01mg/L methylene blue) in an Eppendorf tube, before being subjected to heat-shock at 40°C for 30 minutes in a water bath. After heat-shock treatment, embryos were placed into 28.5°C egg water and incubated until morphological and molecular assessments could be performed. Control *Gt(dmd-citrine)* ^*ct90a*^ embryos were kept at 28.5°C for the entire time as a comparison.

### In situ Hybridization Chain Reaction (HCR)

20 25-mer probes were designed against exon 2 of the *dmd* mRNA; seven 25mer probes were used for the detection of *tropomyosin 3*, and six for the detection of *citrine* (sequences provided in supplemental information). *In situ* HCR was performed as described in [47, 48]. Briefly, zebrafish embryos were fixed in 4% PFA overnight at 4°C, followed by washing in PBS and a series of dehydrations and rehydrations in methanol and PBST. Pre-hybridization was done in 50% hybridization buffer (HB; 50% formamide, 2X Saline Sodium Citrate (SSC), 9mM citric acid, 0.1% Tween-20, 500 μg/mL tRNA and 50 μg/mL Heparin) for 30min at 55°C. Hybridization was performed overnight in 50% HB with 6pmol of each probe at 55°C. The next day the specimens were washed in a series of 50% HB and 2X SSC, followed by a series of 2X SSC (300mM NaCl, 30mM Na3Citrate-2H_2_0) and PBST washes. The samples were pre-hybridized in 40% HB (40% formamide, 2X SSC, 9mM citric acid pH6, 0.5% Tween 20, 2.5 mg/mL tRNA, 250 ug/mL Heparin) for 30min at 45°C and the fluorescently labelled hairpins were snap-cooled before use. After overnight incubation at 45°C with the hairpins, the embryos were washed and mounted for imaging. For counterstaining of the nuclei, 1ug/mL of DAPI in PBS was added to samples after in HCR reaction was completed and allowed to incubate at room temperature for 1hr before imaging.

## Results

### Identification of a gene trap in the *dmd* locus

**Fig 1:**
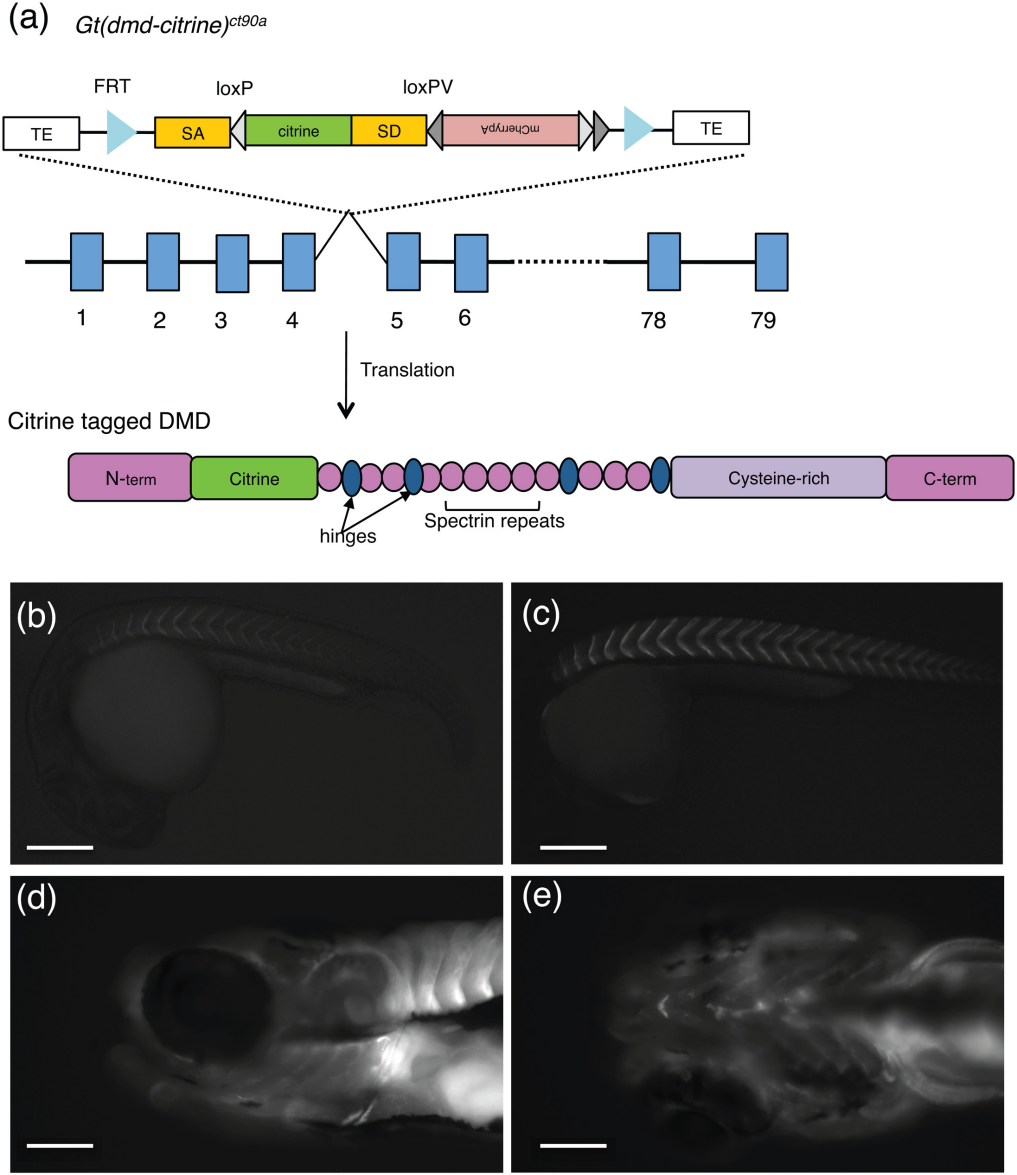
FlipTrap *Gt(dmd-citrine)* ^*ct90a*^ line allows visualization of Dmd protein. (a). Schematic of the FlipTrap vector inserted within *dmd* locus. Citrine insertion (green) occurs within intron 4–5 in *dmd* in the *Gt(dmd-citrine)* ^*ct90a*^ trap line (top) and upon translation produces a fluorescently tagged full-length functional Dmd protein (bottom). The exons (blue rectangles), introns (blank line) and domains of the protein are not drawn to scale. (b-e). Wide-field fluorescent images of Dmd-Citrine expression in the *Gt(dmd-citrine)* ^*ct90a*^ line, in the trunk skeletal muscles (b-c) at 24hpf (b) and 32hpf (c); (d-e). Expression in the cranial skeletal muscle at 6dpf (lateral (d) and ventral (e) views). Ventral view showing expression in the skeletal muscle of the branchial arches. Scale bars: (b)-(c) 100μm, (d)-(e) 25μm.

From a gene trap screen in which the Tol2-based FlipTrap vector was randomly integrated into the zebrafish genome, we isolated a line (*ct90aGT*) that exhibited expression in the developing muscle (Fig 1b–1e). The FlipTrap vector contains an internal exon encoding a variant of yellow fluorescent protein, citrine [42]. When integrated into the intron of actively expressed genes, citrine is transcribed as an artificial exon to create a fusion transcript with the trapped locus. Translation of the trapped transcript leads to expression of a full-length Citrine fusion protein (Fig 1a). In the *ct90aGT* line, the Citrine fusion protein expression is visible starting at 18 hpf, by wide-field and confocal microscopy, in the somite boundaries (Fig 1b–1e). This expression increases over developmental time as indicated by an increase in Citrine fluorescence (Fig 1b–1e). By 6dpf, Citrine fusion protein expression was detected in all skeletal muscle including cranial muscles (Fig 1d and 1e).

Molecular analysis by 3’ Rapid Amplification of cDNA Ends (3’-RACE) with primers against the citrine sequence [42] revealed that the FlipTrap vector inserted between exons 4 and 5 of the *dystrophin* (*dmd)* locus (Fig 1a). Both exons 4 and 5 are in phase zero, as is the artificial citrine exon, creating an in-frame fusion protein. Protein domain prediction suggests that the gene trap creates a fusion protein with Citrine between the two CH domains in the N-terminal calponin homology domain of Dmd (Fig 1a), and would not be expected to disrupt the function of Dmd. Consistent with this prediction, *Gt(dmd-citrine)* ^*ct90a*^ homozygous individuals are viable and do not exhibit muscular dystrophy as would be expected if the insertion in *dmd* were mutagenic [41]. *Gt(dmd-citrine)* ^*ct90a*^ homozygous individuals are viable into adulthood and show no noticeable morphological or behavioral phenotypes that have been indicative of mutations in *dmd* such as swimming defects. Western blot analysis indicates that the Dmd-citrine fusion protein is expressed as a single protein, approximately 30 KDa larger than the Dmd protein, consistent with the citrine exon’s predicted molecular mass of 30 KDa. While multiple isoforms have been documented for Dmd [49], our data suggest that the gene trap insertion leads to a single isoform being tagged in the *ct90aGT* line.

### *Gt(dmd-citrine)* ^*ct90a*^ recapitulates endogenous Dmd protein expression

**Fig 2:**
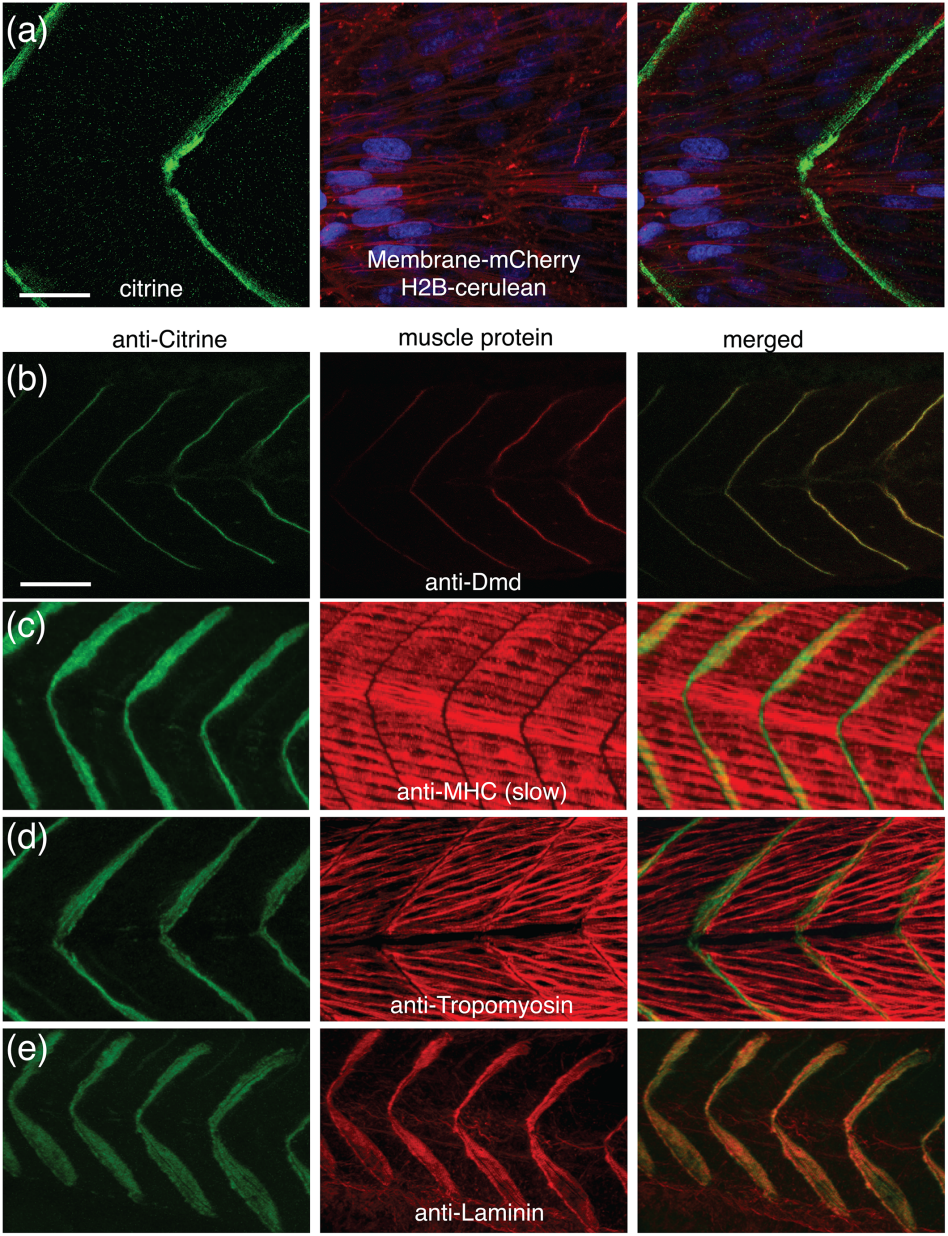
The *Gt(dmd-citrine)* ^*ct90a*^ trap line recapitulates endogenous Dmd protein expression. (a). Single optical section confocal images of live 30hpf *ct90aGT* embryo show that Dmd-citrine fusion protein is localized at the somite border. Dmd is in green (citrine), nuclei are in blue (H2B-cerulean), and membranes are in red (membrane-mCherry mRNA injected). (b). Antibody staining for Citrine and endogenous Dmd in the trap line confirms their co-localization at the somite border. Anti-Citrine label is in green and anti-Dmd label is in red. (c-f). Antibody staining for Citrine and endogenous muscle proteins. Myosin heavy chain (c) and Tropomyosin (d) are expressed in the muscle fibers while Laminin (e) localizes to the extracellular matrix of the myosepta. Scale bar = 10μm (a), 20μm (b).

Expression and co-localization analyses indicate that the *Gt(dmd-citrine)* ^*ct90a*^ line recapitulates the expression and localization of endogenous Dmd protein. First, subcellular localization analysis of Dmd-citrine in *ct90aGT* embryos by co-expression of membrane (membrane-mCherry) and nuclear (H2B-cerulean) labels reveal that Dmd-citrine localizes to the myoseptal junctions at the sarcoplasmic side of the sarcolemma, and is excluded from the interior of the muscle cells (Fig 2a). Second, co-localization analysis by antibody staining for proteins expressed in the either muscle fibers or myosepta indicate that Dmd-citrine co-localizes with myoseptal components (Fig 2b–2e). Myosin heavy chain and Tropomyosin are expressed in the muscle fibers while Laminin localizes to the extracellular matrix of the myosepta [50, 51]. Antibody co-localization experiments performed on these proteins in *ct90aGT* embryos show that Dmd-citrine localizes to the myosepta, similar to Laminin (Fig 2e). Finally, immunohistochemistry with antibodies performed on endogenous Dmd and Citrine in *ct90aGT* embryos show that Dmd and Dmd-citrine colocalize (Fig 2b). The colocalization between Dmd and Dmd-citrine can be detected at the myoseptum starting at 18-19somite, suggesting that there is no difference in the maturation time of Dmd and Dmd-citrine protein. Collectively, these results show that the *Gt(dmd-citrine)* ^*ct90a*^ line accurately reflects Dmd expression in zebrafish embryos.

### Temporal differences in the localization of *dmd* transcript and Dmd protein during somite development

**Fig 3:**
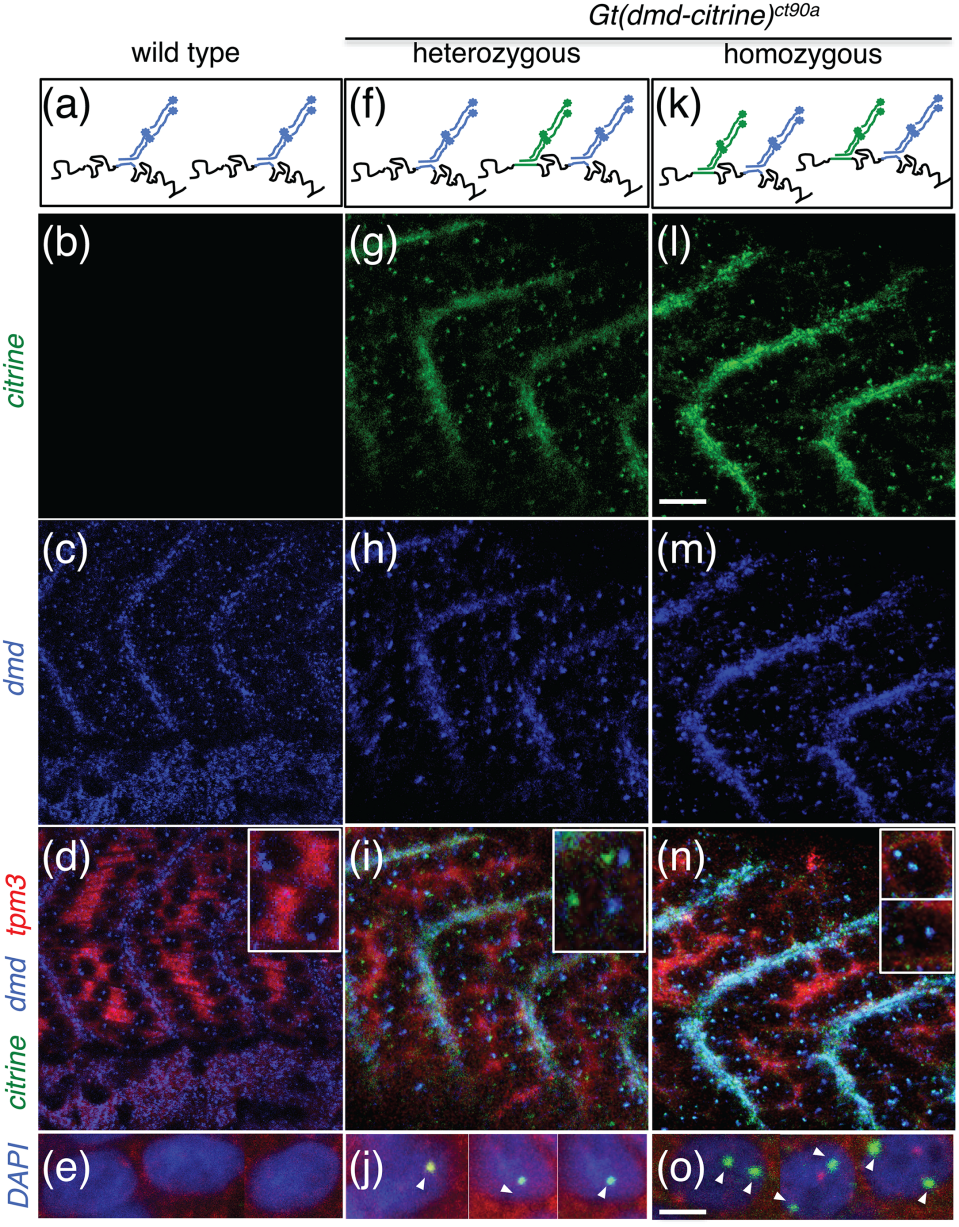
The *Gt(dmd-citrine)* ^*ct90a*^ trap line enables visualizing differential expression in homozygous and heterozygous embryos via in situ HCR. (a, f, k) Schematic of *in situ* HCR experiment showing *dmd* and *citrine* HCR probe binding sites within *dmd* and *dmd-citrine* transcript. The *dmd* HCR probe should bind to both *dmd* and *dmd-citrine* transcripts, while *citrine* HCR probe binds only to *dmd-citrine* transcript. (b-e, g-j, l-o). *in situ* HCR analysis of *dmd* (blue) and *dmd-citrine* (green) transcript in wild type (b-e), heterozygous (g-j) and homozygous (l-o) *Gt(dmd-citrine)* ^*ct90a*^ embryos. *Tmp3* transcripts counter-stain muscles in red. Insets in (d),(i) and (n) show zoomed in view of transcription sites in the nuclei. Two dots are detected in homozygous embryos (k-n) whereas only one dot is detected in the nuclei of heterozygous *ct90aGT* embryos (f-i) while no citrine dots are detected in wild types embryos (a-d), consistent with the copy number of citrine inserted in the respective embryos. (e, j, o) Magnified images of the three types of embryos with nuclei stained in DAPI and the transcripts indicated by arrowheads. Scale bar (b-d, g-i, l-n)20μm (e,j,o)10μm.

*Dmd* transcript has been reported to be spatially distinct in both mature and developing muscles. In human skeletal muscles, *dmd* transcripts are localized preferentially to the sub-sarcolemma of muscle cells, both in normal and DMD/BMD patients [52]. In contrast, in developing zebrafish muscle, *dmd* mRNA is initially detected throughout the cytoplasm of myoblasts and localizes to the transverse myosepta by 24hpf [32, 36, 53]. To assess the localization of *dmd* mRNA and determine if the *dmd-citrine* transcript recapitulates the endogenous localization of *dmd*, we employed *in situ* Hybridization Chain Reaction (HCR) [48] to simultaneously detect *dmd* and *dmd-citrine* transcripts in *ct90aGT* embryos. The *dmd* HCR probes bind to both *dmd* and *dmd-citrine* transcripts, while the *citrine* probes bind only to *dmd-citrine* transcripts (Fig 3a–3n). Using this combination of probes, we found that *dmd* and *dmd-citrine* fusion transcripts both localize predominantly to the transverse myosepta in 48hpf embryos (Fig 3g–3i and 3l–3n). Additionally, *dmd* and *dmd-citrine* probes localize as dots in the nuclei suggesting that the HCR probes are labeling nascent transcripts (Fig 3i, 3j and 3n–3o). The citrine probe labels a single dot in heterozygous *ct90aGT* embryos and two dots in homozygous embryos; the *dmd* probe labels two dots in all embryos (Fig 3i and 3n inset, Fig 3j and 3o, S1 Fig). These data are consistent with heterozygous *ct90aGT* embryos having a single copy of the gene trap and homozygous *ct90aGT* embryos having two copies. More importantly, the *dmd* and *citrine* probes show colocalization at the myosepta and in the nuclei in homozygous embryos indicating that *dmd-citrine* recapitulates *dmd* transcript localization.

**Fig 4:**
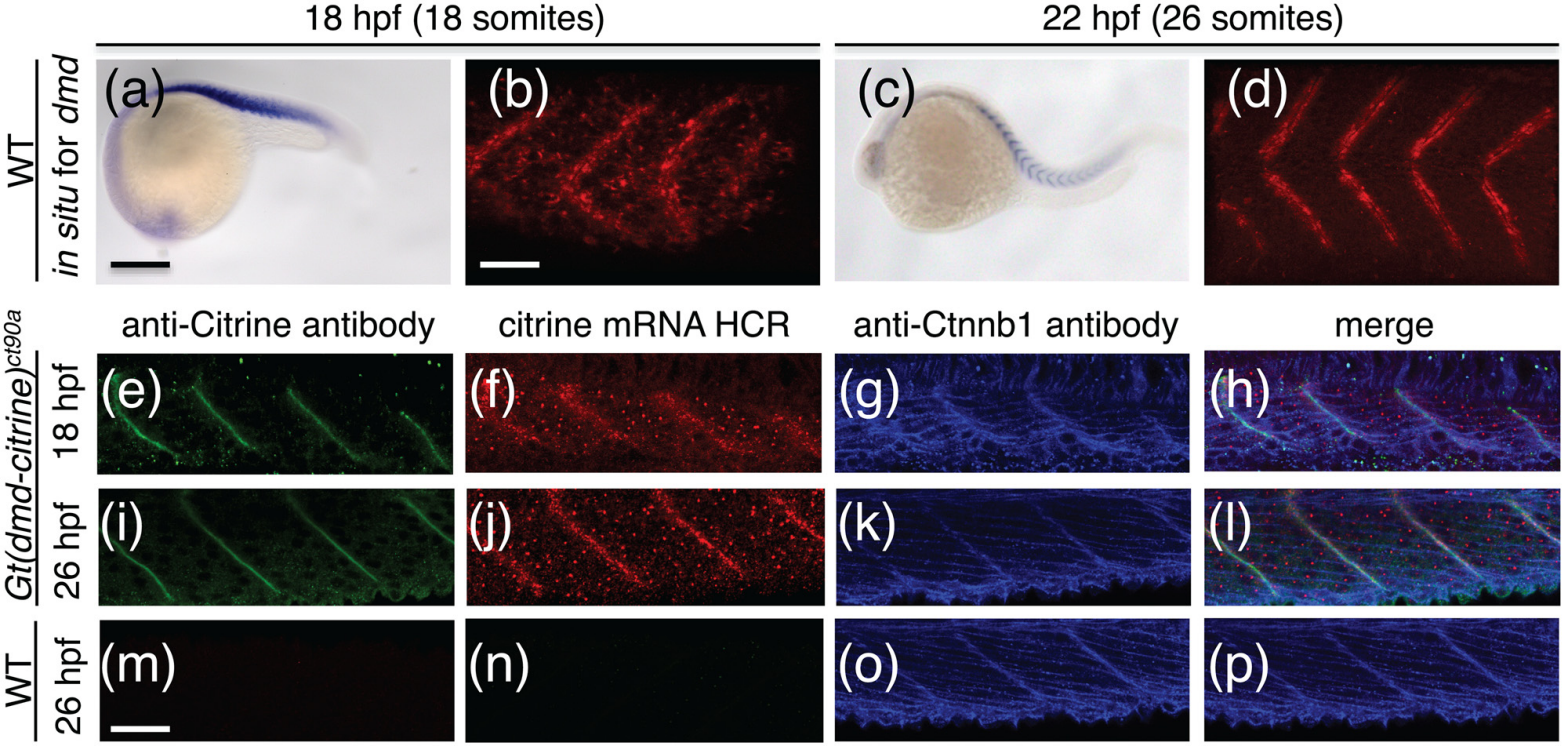
*dmd* mRNA and Dmd protein expression show differences in spatial expression. (a-d). Chromogenic *in situ* hybridization for *dmd* in wild type embryos at 18hpf (a,b) and 22hpf (c,d). (a,c) Wide field image of 18hpf and 22hpf embryos stained for *dmd* expression. (b,d) 3D projections of confocal z-stack of embryos in (a) and (c), respectively. Fluorescent signal of NBT/BCIP stain for *dmd* transcript reveals spatial confinement of transcript between somites. (e-p). Confocal image of *dmd*-*citrine* transcript (f,j,n) detected by *in situ* HCR and antibody staining for DMD-citrine protein (e,i,m) in *Gt(dmd-citrine)* ^ct90a^ (e-l) and wildtype (m-p) embryos at 18hpf and 26hpf. Counter-stain with antibody to Ctnnb1 in blue (g,k,o). (h,l,p) Merged of image of (e-g, i-k, and m-o). Comparison of protein and transcript expression at 18hpf (e-h) and 26 hpf (i-l) in *Gt(dmd-citrine)* ^ct90a^ embryos show that DMD protein localize exclusively to the myosepta while *dmd-citrine* mRNA is expressed in the cytoplasm at 18hpf and becomes more localized to the myosepta at 26hpf. Dmd-citrine expression in the nucleus can be seen as dots that appear similar in distribution between 18hpf and 26hpf. (m-p) DMD-citrine protein (m) and transcript (n) are not detected in wildtype embryos. Anti-Citrine antibody staining is in green, *citrine* mRNA HCR is in red and anti-Ctnnb1 antibody is in blue. Scale bars (a) 50μm (b,m) 20 μm.

To further characterize the localization of *dmd* transcript, we performed a time-course of *dmd* expression using chromogenic *in situ* hybridization. Assessing the expression of *dmd* in one hour developmental time intervals starting at 19.5 hpf, we found that *dmd* transcript switches from a non-localized diffused pattern in the cytoplasm of the muscle cells at 21.5hpf, to a restricted pattern at the transverse myosepta at 22.5 hpf (Fig 4a–4d), where it remains throughout development. The same switch from a diffused to localized pattern is observed when detecting *dmd* transcript by HCR *in situ* hybridization (S2 Fig), with the exception that *in situ* HCR also detects the nuclear dots in all developmental stages.

The differential distribution of *dmd* transcript led us to ask whether *dmd* protein exhibits a similar change in localization during development. To assess mRNA and protein in the same sample, we performed *in situ* HCR to detect *dmd* transcript followed by immunohistochemistry to detect Dmd protein with an antibody to Citrine (Fig 4e–4l). This dual detection approach revealed that Dmd protein is restricted to the myosepta from the onset of Dmd protein expression, at 18 hpf, when *dmd* transcript is diffusely dispersed in the cytoplasm (Fig 4e–4h). By 26 hpf both *dmd* transcript and protein are localized to the myosepta with nascent *dmd* transcripts detected as small dots in nuclei of muscle fibers (Fig 4i–4l)).

### Dynamics of Dmd protein localization during somitogenesis

**Fig 5:**
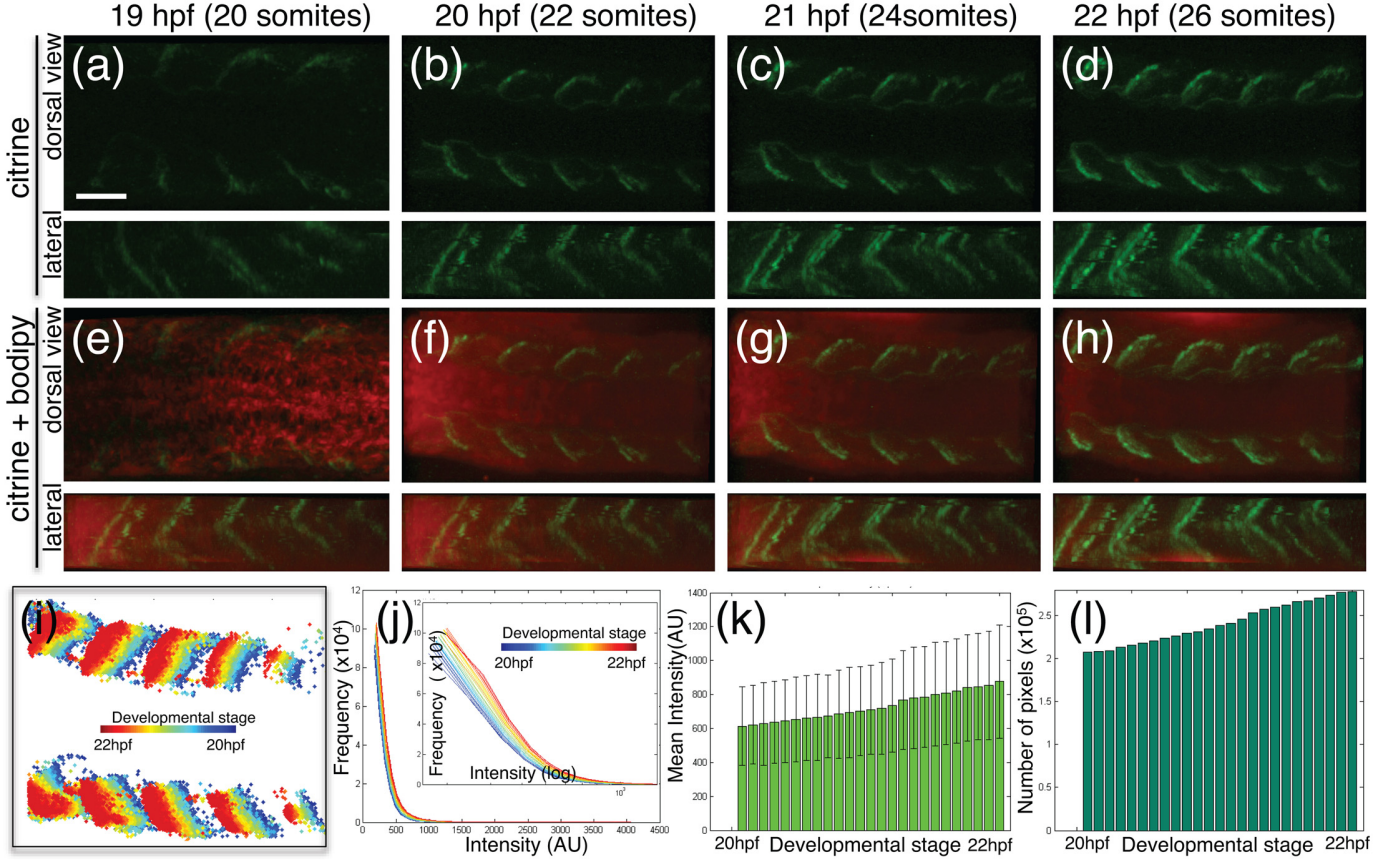
Dmd Protein expression increases both in intensity and area over development. (a-h). 3D projections of confocal Z-stacks images in developing trunk of *Gt(dmd-citrine)* ^*ct90a*^ embryos from the 20 to 26 somites stages (19–22 hpf), showing expression of Dmd-citrine (a-d, green) and the vital stain BodipyTR methyl ester (e-h, red) from dorsal and lateral view. (i-l) Quantitation of Dmd-citrine fluorescent signal in confocal time-lapse. (i). Kymograph of Dmd-citrine expression color-coded according to developmental stage. The spatial shift in expression appears to be due to tissue growth and expression enhancement over time. (j). Histogram of fluorescent pixel intensities color-coded according to developmental stage with intensity in X-axis and frequency in Y-axis. Plot shows Dmd-citrine expression increasing over time resulting in shift slope along the X-axis (blue to red). The inset shows the same plotted on a log scale to enhance observation. (k). Mean pixel intensity plotted against development stage showing increase in fluorescent intensity of Dmd-citrine as development progresses. The error bars denote the variation among all the pixels at that particular stage. (l). Total pixel count of Dmd-citrine fluorescent plotted against developmental time showing that Dmd-citrine expression increase in spatial area over time. Scale bar = 20μm.

The Citrine fluorescent signature in the *ct90aGT* line enables real-time analysis of Dmd localization during somitogenesis (Fig 5a–5h); S3 Fig, S1 Movie). Dmd-citrine could be detected at low levels in the somite boundaries starting at 18–18.5 hpf (Fig 5a and 5e). The expression increases in intensity from the anterior to posterior somites (Fig 5a–5h). In vertebrates, the somatic muscles develop from the paraxial mesoderm in an anterior-to-posterior wave. In zebrafish, this anterior-to-posterior developmental pattern initiates after gastrulation (12 hpf) during which *dmd* transcripts have been detected [36]. However, we detect Dmd-citrine protein localization at the somite boundaries 6 hours after transcripts can be detected, at 18 hpf, indicating that there is a significant delay between transcriptional active and protein maturation.

To quantify the spatio-temporal dynamics of Dmd-citrine expression, we mapped the pixel intensity of the fluorescence signal over time (Fig 5i–5l). Kymographs of the Dmd-citrine expression show that the Dmd-citrine protein shifts in space over time (Fig 5l). However, fluorescent images indicate that Dmd-citrine expression remains at the myoseptum (Fig 5a–5h) suggesting that the shift in space may be due to movement of the tissue or growth of the embryo. The shift in Dmd-citrine expression is unlikely due to an increase in the overall size of the somites, as the width of each somite remains constant and the distance between each myoseptum remains the same (Fig 5a–5l). The shift in Dmd-citrine protein expression is accompanied by an increase in the total area in which Dmd-citrine can be detected in each developing somite (Fig 5i, 5k and 5l), indicating that more Dmd-citrine proteins are accumulating at the myoseptum over time. To quantify the relative levels of Dmd-citrine in time, we mapped the fluorescent intensity from the time-lapse analysis. We find that the relative mean pixel intensity increases over developmental time, indicating that more fluorescent signal can be detected per unit area (Fig 5k). Additionally, more pixels have fluorescent signature over developmental time (Fig 5l), indicating that as the somites mature more Dmd-citrine proteins are localizing to the myosepta.

### Using the *Gt(dmd-citrine)* ^*ct90a*^ line for phenotyping muscular dystrophy

**Fig 6:**
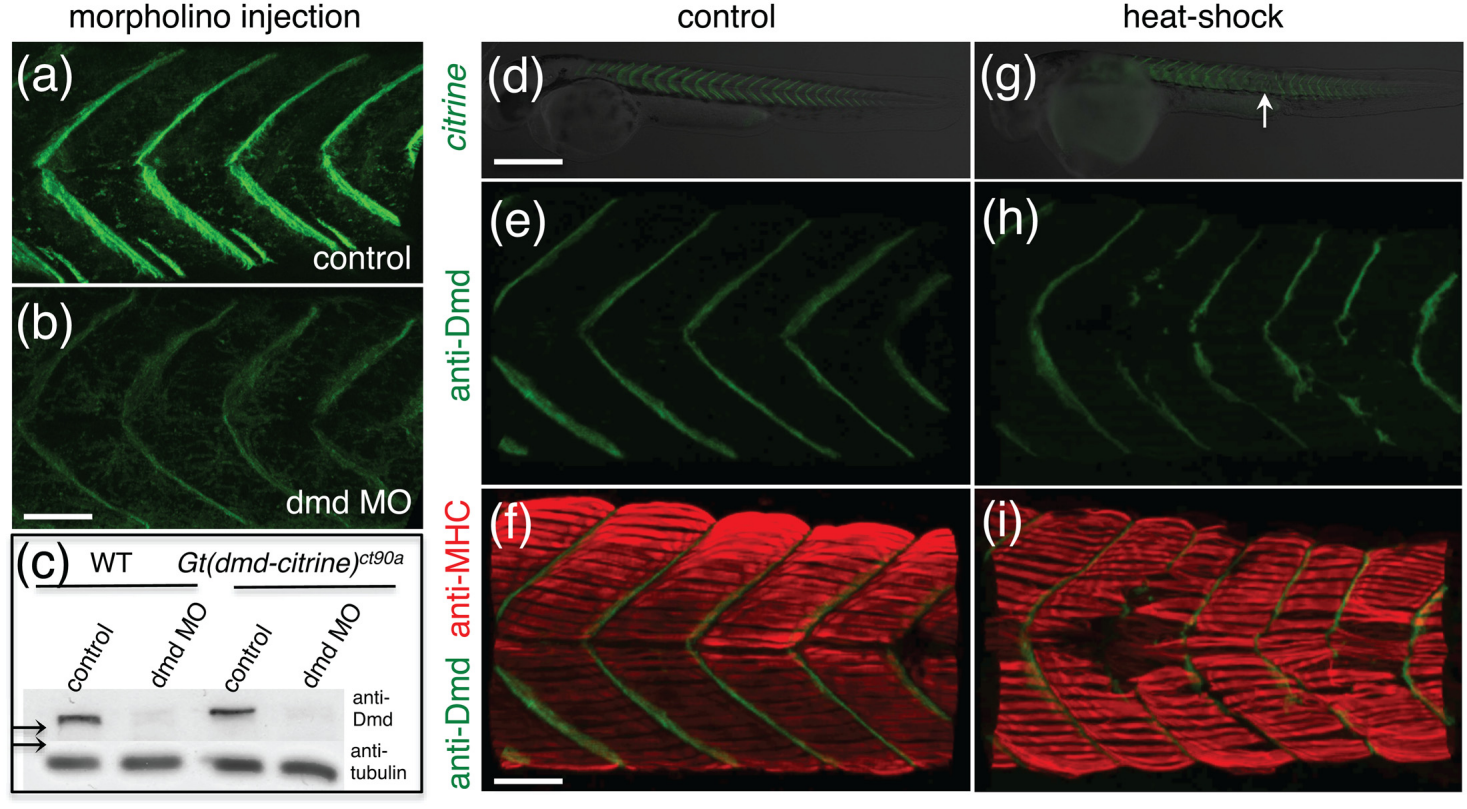
Phenotyping of muscular dystrophy with *Gt(dmd-citrine)* ^*ct90a*^. (a,b). Dmd-citrine expression in control (a) and *dmd* morphants (b). Embryos in (a) and (b) were imaged with the same laser power and gain settings. (c) Western-blot of protein extracts from WT (lanes 1, 2) and *Gt(dmd-citrine)* ^*ct90a*^ (lanes 3, 4) embryos with antibody to DMD and tubulin (loading control). Embryos for protein extracts were injected with control morpholino (lanes 1 and 3) and *dmd* morpholino (lanes 2 and 4, n = 3–6). Arrows point to size difference between Dmd and Dmd-citrine. (d,e) Widefield fluorescent image of 32hpf *ct90aGT* embryos untreated (d) and treated with heat-shocked at 6-somites stage. Arrow point to defects in Dmd-citrine expression visible between somites 16–25. (f-i) Heat-shocked embryos stained for Dmd (f,g; green) and anti-MHC (h,i; red) at 32hpf. Scale bars (d,e) 50μm (f-i) 20 μm.

The ability to detect Dmd protein *in vivo* enables the use of the *Gt(dmd-citrine)* ^*ct90a*^ line to assess muscle phenotypes. First, we asked how morpholino anti-sense knockdown of Dmd would affect Dmd-citrine expression in *ct90aGT* embryos. Injection of a previously published ATG morpholino against *dmd* [45] resulted in a significant decrease in the amount of Dmd-citrine fluorescence detected in *ct90aGT* embryos (Fig 6a and 6b) compared to control. The decrease in Dmd-citrine fluorescence correlated with a reduction of Dmd protein detected by Western-blot (Fig 6c). In addition, *ct90aGT* morpholino knockdown embryos exhibited dystrophy phenotype in which the muscles fibers are disorganized, the sarcolemma borders are not precisely defined and the muscle fibers vary in size (data not shown).

In addition to morpholino knockdown, we asked whether the *ct90aGT* line could be used to assess other muscular dystrophy phenotypes. Heat-shocks given during somitogenesis have been shown to result in somite abnormalities, with defects appearing later than the given treatment and not affecting somite formation at the time of the shock [54–56]. We performed heat-shock on *ct90aGT* embryos to determine the affects of heat-shock on Dmd-citrine expression. Performing a single 30-minute heat-shock treatment in 6-somite stage (12 hpf) embryos from the *Gt(dmd-citrine)* ^*ct90a*^ line resulted skeletal muscle defects between somites 16–25 at 32 hpf (Fig 6d–6i). In the defective somites, disorganized muscle fibers are accompanied by an absence of Dmd-citrine localization at the myoseptum (Fig 6g and 6i). This phenotype is consistent with Dmd-citrine role as structural protein important linking the skeletal muscle to the extracellular matrix.

## Discussion

We describe a zebrafish gene trap line, *Gt(dmd-citrine)* ^*ct90a*^ that expresses a Dmd-citrine protein fusion allowing the dynamic study of muscular dystrophy. We have shown that the expression of Dmd-citrine in these embryos recapitulates endogenous Dmd protein and mRNA localization. The tagging of Dmd with citrine did not seem to affect the interaction of Dmd with its DAPC partners. Defects in protein interactions within the DAPC would have lead to dystrophic phenotypes, and we did not observe any phenotypes in homozygous *Gt(dmd-citrine)* ^*ct90a*^ embryos. This transgenic line enables spatio-temporal study of Dmd dynamics in a live vertebrate animal model.

*dmd* transcript has been described to exhibit spatial differences in localization during development. We have found that *dmd* transcript transitions from a cytoplasmic localization in the myoblast to a discrete localization pattern in the myoseptum between 21.5 hpf and 22.5 hpf (Fig 4(a)–4(d)). This transition in localization pattern coincides with a developmental stage when myoblasts fuse to form myotubes [57]. The syncytial nature of the myotubes may allow *dmd* transcripts to be discretely localized to the myoseptum. In the individual myoblast, the cell membrane may serve as a physical barrier for localization to the myosepta. Consistent with this hypothesis, we observe localization of *dmd* transcript in the myoseptum in 21.5 hpf embryos when cytoplasmic *dmd* transcript could be detected, indicating that localization of the transcript can occur at these earlier stages in development.

Quantitative analysis of the time-lapse data shows that not only does the mean signal intensity of Dmd-citrine increase (by ~ 45%) as development proceeds but the total expression area near the myoseptum increases as indicated by a 40% increase in the total number of pixels (Fig 5l). These results suggest that, as the myoblasts transitions to syncytial fibers, more Dystrophin is recruited to the myosepta. The accumulation of Dmd to the myosepta, as the muscle fibers mature, hints towards its importance in providing the mechanical anchoring for contracting muscle. Consistent with a role in anchoring muscle fibers, morpholino knockdown of Dmd show a progressive loss of Dmd-citrine expression and defects in muscle integrity. Additionally, heat shock experiments that result in discrete muscle integrity defects between somites 15–25 also results in lost of Dmd-citrine localization at these disrupted somites.

Interestingly we found that, during early zebrafish embryo development, *dmd* mRNA transcripts first appeared diffused in the cytoplasm before being localized at the myosepta. We did not observe such a differential pattern for Dmd protein. Dmd-citrine is initially detected at the myoseptum between the 18–19 somite stages prior to the localization of the mRNA at the myoseptum. This temporal difference in the localization of the mRNA and protein to the myosepta suggests that DMD-citrine is translated or matures into detectable fluorescence at the myoseptum. Immunohistochemistry with antibodies to Dmd suggests that the temporal maturation of Dmd and Dmd-citrine are similar as both proteins are detected at the myoseptum starting at the 18–19 somite stage. At this stage cell fusion should be completed in the anterior somites, but is not yet complete in the posterior somites [58]. Consistent with this anterior to posterior wave of maturation of the somites, we observe a similar anterior to posterior wave of localization of Dmd-citrine to the myoseptum in the time-lapse microscopy (Fig 5, S4 movie).

As development progresses, we see significant decrease in cytoplasmic distribution of *dmd* transcript over 4 hours from 18hpf to 22hpf. *In situ* HCR shows the nascent transcript sites in the nucleus with nearly no localization of dmd-citrine or *dmd* mRNA in the cytoplasm by 26hpf when most of the myoblasts have completed fusion (Fig 4, S2 Fig). There is a possibility that Dmd protein is present in the cytoplasm at levels below our detection limit; however, our data indicate that the majority of the Dmd protein are localized to the myosepta. Previous work on dynamics of mRNA-protein (mRNP) nucleocytoplasmic transport in mammalian cells has shown that the cytoplasmic diffusion coefficient of dystophin mRNAs is low [59]. It would be of interest to assess the dynamics of Dmd protein that is predicted to be slower given it’s large size (427 KDa protein). Therefore increasing localization of the transcript itself at the myoseptal junctions might facilitate effective translation or maturation of Dmd-citrine into detectable fluorescence at the site of its activity. This localization might be helpful in bypassing the time required for protein diffusion from the cytoplasm of fusing myoblasts or mature myotomes to the somite boundaries, thereby making the availability of Dystrophin, for anchoring, limited only by the rate of on-site translation. The *ct90aGT* line provides opportunities for future studies to test these hypotheses.

## Supporting Information

S1 Fig Co-localization of dmd and dmd-citrine transcript in *Gt(dmd-citrine)* ^*ct90a*^ embryos *via in situ* HCR. (a-c) *in situ* HCR of *dmd* (red), *citrine* (green) and *tpm3* in *Gt(dmd-citrine)* ^*ct90a*^ embryos. *Tpm3* labels the cytoplasm of the myocytes while both *dmd* and *citrine* are detected at the myosepta and in the nucleus. *Dmd* and *citrine* transcripts show overlap expression at the myosepta. (d) Merge of (a-c).
(EPS)

S2 Fig Differential localization of *dmd* transcript over developmental time as detected via *in situ* HCR. (a) *in situ* HCR of *dmd-citrine* (red) in *ct90aGT* embryos counter-stained for *tpm3* (blue). *tpm3* Counter-stain show the nuclei by negative contrast. Inset shows zoomed in view of active transcription sites in the nuclei. (b-d) 3D projections of confocal z-stacks of *dmd* transcripts stained by *in situ* HCR at 21hpf (b), 22hpf (c), and 25hpf (d). *dmd* transcript transitions from the entire somite to the somite boundaries as development progresses.
(EPS)

S3 Fig Time lapse imaging of Dmd-citrine expression in *Gt(dmd-citrine)* ^*ct90a*^ trap line. 3D projections of confocal Z-stacks images in developing trunk of *Gt(dmd-citrine)* ^*ct90a*^ embryos from the 18 to 35 hpf, showing expression of Dmd-citrine (green) and the vitalstain BodipyTR methyl ester (red) from dorsal view. The expression of Dmd-Citrinefusion (green) increases with development. The microscope gain has been kept high inorder to detect citrine expression as early as possible. Images taken from frames withinSupplemental movie 1. Scale bar = 20μm
(EPS)

S1 Movie Time-lapse of Dmd-citrine. Time-lapse movie from confocal z-stack projections of Dmd-citrine (green) expression in*Gt(dmd-citrine)* ^*ct90a*^ embryo stained with BodipyTR methyl ester from 18 to 35 hpf. Dorsal view image.
(MOV)

S1 Text Methods. (DOCX)
