# Supplementary material for: Spatio-Temporal Differences in Dystrophin Dynamics at mRNA and Protein Levels Revealed by a Novel FlipTrap Line
Source: PLoS One. 2015 Jun 17;10(6):e0128944. doi: 10.1371/journal.pone.0128944 (PMC4471274; doi:10.1371/journal.pone.0128944)
Supplement: S1 Text — (DOCX) [file pone.0128944.s005.docx]

Supporting Information

**Spatio-temporal differences in dystrophin dynamics at mRNA and protein levelsrevealed by a novel FlipTrap line**

Frederique Ruf-Zamojski^1,2*^, Vikas Trivedi^1*^, Scott E. Fraser^1,3^, and Le A. Trinh^1,3^

Supplementary Figure Legends

**S1 Fig. Co-localization of dmd and dmd-citrine transcript in *Gt(dmd-citrine)^ct90a^* embryos *via in situ* HCR.**

(a-c) *in situ* HCR of *dmd* (red), *citrine* (green) and *tpm3* in *Gt(dmd-citrine)^ct90a^* embryos. *Tpm3* labels the cytoplasm of the myocytes while both *dmd* and *citrine* are detected at the myosepta and in the nucleus. *Dmd* and *citrine* transcripts show overlap expression at the myosepta. (d) Merge of (a-c).

**S2 Fig. Differential localization of *dmd* transcript over developmental time as detected via *in situ* HCR.**

(a) *in situ* HCR of *dmd-citrine* (red) in *ct90aGT* embryos counter-stained for *tpm3* (blue). *tpm3* Counter-stain show the nuclei by negative contrast. Inset shows zoomed in view of active transcription sites in the nuclei. (b-d) 3D projections of confocal z-stacks of *dmd* transcripts stained by *in situ* HCR at 21hpf (b), 22hpf (c), and 25hpf (d). *dmd* transcript transitions from the entire somite to the somite boundaries as development progresses.

**S3 Fig. Time lapse imaging of Dmd-citrine expression in *Gt(dmd-citrine)^ct90a^* trap line.**

3D projections of confocal Z-stacks images in developing trunk of *Gt(dmd-citrine)^ct90a^* embryos from the 18 to 35 hpf, showing expression of Dmd-citrine (green) and the vitalstain BodipyTR methyl ester (red) from dorsal view. The expression of Dmd-Citrinefusion (green) increases with development. The microscope gain has been kept high inorder to detect citrine expression as early as possible. Images taken from frames withinSupplemental movie 1. Scale bar= 20𝜇m

**S1 Movie**: **Time-lapse of Dmd-citrine.**

Time-lapse movie from confocal z-stack projections of Dmd-citrine (green) expression in*Gt(dmd-citrine)^ct90a^* embryo stained with BodipyTR methyl ester from 18 to 35 hpf. Dorsal view image.

**S1 Text**

Methods

HCR probes

Target mRNA: *tropomyosin 3* (*tpm3*) – (Amplifier HCR2, Alexa fluorophore 488)Initiator / Spacer / ProbeSequences

1.CCgAAUACAAAgCAUCAACgACUAg/AAAAAA/UCCUCAACCAgCUggAUACgCCUgUUCAgAgAAgCCACCUCUgCCUCAgC

2.CCgAAUACAAAgCAUCAACgACUAg/AAAAAA/CCAgCUUUUgCAgggCUgUggCCAgUCUCUCCUgAgCACgAUCCAACUCC

3.CCgAAUACAAAgCAUCAACgACUAg/AAAAAA/AAUCACCUUCAUCCCUCUCUCgCUCUCAUCUgCggCCUUCUCggCUUCCU

4.CCgAAUACAAAgCAUCAACgACUAg/AAAAAA/UggAUCUCCUgCAgCUCCAUCUUCUCCUCAUCCUUCAgAgCCCUgUUCUC

5.CCgAAUACAAAgCAUCAACgACUAg/AAAAAA/CUUCAUAUUUgCggUCAgCCUCCUCAgCAAUgUgCUUggCCUCCUUAAgC

6.CCgAAUACAAAgCAUCAACgACUAg/AAAAAA/CUCUgUACgCUCCAACUCUCCCUCAACgAUCACCAgCUUACgAgCCACCU7.CCgAAUACAAAgCAUCAACgACUAg/AAAAAA/UggUUUUCUCCAgUUUggCCACAgACCUCUCAgCAAACUCUgCACgggUC

Target mRNA: *dystrophin* (*dmd*) – (Amplifier HCR2, Alexa fluorophore 647) Initiator / Spacer / Probe Sequences

1.CCgAATACAAAgCATCAACgACTAg/AAAAAA/AgCTgCTCCTCAgAgAAATgCTgCCAgCACAgCAggATCTTTTgAAggAg

2.CCgAATACAAAgCATCAACgACTAg/AAAAAA/ggATggACTggACCAACTCCTCCTTTTgAgTCAgCCAAgAATCAAACAgC

3.CCgAATACAAAgCATCAACgACTAg/AAAAAA/CTTCCgCAggTTggCggCTACTTCATTTgggTCATTTgTgCCACTgCTTT

4.CCgAATACAAAgCATCAACgACTAg/AAAAAA/TTgTCCATggTCTgCCTCCTCAgCTCCAggTCTgCTTTTAAAATCgCCAg

5.CCgAATACAAAgCATCAACgACTAg/AAAAAA/CCTCTTTgCTCTTgATgTTAgTCAgCAgATCCTggACgAgCgAgCAAAgC

6.CCgAATACAAAgCATCAACgACTAg/AAAAAA/ATTTgTCCCAgCgCTgAgCAAACCTCTCCAgCTTTgCTTCCAgCTTCCCA

7.CCgAATACAAAgCATCAACgACTAg/AAAAAA/ggTgACAATAgTTgAAATCTTggTgCTggTgAgCTgAAgCgCCTgCACCA

8.CCgAATACAAAgCATCAACgACTAg/AAAAAA/ACCTTggTgACAgTTgCCATggTTgCgTgCgTgATCTCCgACTgggATgT

9.CCgAATACAAAgCATCAACgACTAg/AAAAAA/TggACATgCCCTCCTTAgTATgCTTCACCATCTTCTTCTggTTCgTggTC

10.CCgAATACAAAgCATCAACgACTAg/AAAAAA/TCCgCAgTTCTgAATCCACgACAATCTgTCTCTTTTTCTgAggTggAggg

11.CCgAATACAAAgCATCAACgACTAg/AAAAAA/gAgTTCTgCCATCTTTgggTggTCCCAACATgTTgTTTgggTCTggTgAT
12.CCgAATACAAAgCATCAACgACTAg/AAAAAA/AAACAgAgggCTTTCTgCATTCgTCTgAgCTTCATTgCCgTCCTgTATgC
13.CCgAATACAAAgCATCAACgACTAg/AAAAAA/TTTgAgATTgTgCTgCTCAAAggCTTCACAggCTgCAggCATgCTCAgAA
14.CCgAATACAAAgCATCAACgACTAg/AAAAAA/ATgCTggTCAgACAgTTgATCACCTgCACgATgTCCATgAACTgCTCgTT
15.CCgAATACAAAgCATCAACgACTAg/AAAAAA/TAAACgTTgAgCAgCCAgTTgAgACACATgTCCACACAgAgAggCACgTT
16.CCgAATACAAAgCATCAACgACTAg/AAAAAA/TgATTCAgACTCTggCAgTAgTgCTggATCAgCAgATgCTCATCATCCCT
17.CCgAATACAAAgCATCAACgACTAg/AAAAAA/TCTCTCCAgCTCTCCCTTCTCTTCAgTCTCCATTgAgATgAggATCTgAg
18.CCgAATACAAAgCATCAACgACTAg/AAAAAA/ACgATCgTACTCCgCTTgCAgCTTCCTgTTTTCCTgCTCCAgATCATTgA
19.CCgAATACAAAgCATCAACgACTAg/AAAAAA/AATCgTCCCTTgTgTTgCCgCAgTAgTTTggCTTCTgCgATTAgTTCTgC
20.CCgAATACAAAgCATCAACgACTAg/AAAAAA/CTgCgATTCCAgCTgCTTgTTgTgATCCTCCAggATTTgCATTCTTgCCT

Target mRNA: *citrine* (Amplifier HCR3, Alexa fluorophore 546) Initiator / Spacer / Probe Sequences

1.TACgCCCTAAgAATCCgAACCCTATg/AAATA/gAACAgCTCCTCgCCCTTgCTCACTCCggATAACTTCgTATAATgTATgC

2.TACgCCCTAAgAATCCgAACCCTATg/AAATA/TTgCCggTggTgCAgATgAACTTCAgggTCAgCTTgCCgTAggTggCATC

3.TACgCCCTAAgAATCCgAACCCTATg/AAATA/TTgAAgAAgTCgTgCTgCTTCATgTggTCggggTAgCgggCgAAgCACAT

4.TACgCCCTAAgAATCCgAACCCTATg/AAATA/TCTTgTAgTTgCCgTCgTCCTTgAAgAAgATggTgCgCTCCTggACgTAg

5.TACgCCCTAAgAATCCgAACCCTATg/AAATA/ATgTTgCCgTCCTCCTTgAAgTCgATgCCCTTCAgCTCgATgCggTTCAC

6.TACgCCCTAAgAATCCgAACCCTATg/AAATA/TgATATAgACgTTgTggCTgTTgTAgTTgTACTCCAgCTTgTgCCCCAgg

7.TACgCCCTAAgAATCCgAACCCTATg/AAATA/TgTTgTggCggATCTTgAAgTTCACCTTgATgCCgTTCTTCTgCTTgTCg

8.TACgCCCTAAgAATCCgAACCCTATg/AAATA/TTCTCgTTggggTCTTTgCTCAgggCggACTggTAgCTCAggTAgTggTT
